# The role of platelet mediated thromboinflammation in acute liver injury

**DOI:** 10.3389/fimmu.2022.1037645

**Published:** 2022-10-27

**Authors:** Sean M. Morris, Abhishek Chauhan

**Affiliations:** ^1^ The Liver Unit, University Hospitals Birmingham, Birmingham, United Kingdom; ^2^ Institute of Immunology and Immunotherapy, University of Birmingham, Birmingham, United Kingdom

**Keywords:** platelet, innate immunity, liver failure- therapy, acute on chronic liver failure (ACLF), ALF, liver, thromboinflammation, , immunothrombosis

## Abstract

Acute liver injuries have wide and varied etiologies and they occur both in patients with and without pre-existent chronic liver disease. Whilst the pathophysiological mechanisms remain distinct, both acute and acute-on-chronic liver injury is typified by deranged serum transaminase levels and if severe or persistent can result in liver failure manifest by a combination of jaundice, coagulopathy and encephalopathy. It is well established that platelets exhibit diverse functions as immune cells and are active participants in inflammation through processes including immunothrombosis or thromboinflammation. Growing evidence suggests platelets play a dualistic role in liver inflammation, shaping the immune response through direct interactions and release of soluble mediators modulating function of liver sinusoidal endothelial cells, stromal cells as well as migrating and tissue-resident leucocytes. Elucidating the pathways involved in initiation, propagation and resolution of the immune response are of interest to identify therapeutic targets. In this review the provocative role of platelets is outlined, highlighting beneficial and detrimental effects in a spatial, temporal and disease-specific manner.

## 1 Introduction

Acute liver inflammation as a result of hepatic injury can occur both in patients with and without pre-existent liver disease. Acute failure (ALF) occurs in the former, specifically when a patient with no antecedent history of liver disease develops significant liver dysfunction due to an acute hepatocellular insult (1). ALF is characterized by deranged liver biochemistry, jaundice, coagulopathy and encephalopathy (1). Viral hepatitides are the leading cause of ALF in the Asia and Africa whilst in the West the majority of ALF due to drug-induced liver injury [acetaminophen (APAP) overdose] (1). Treatments options for APAP-induced ALF are limited (2) and treatment is therefore based largely around supportive care often in an intensive care setting; ultimately liver transplantation may be required (1). Ten percent of liver transplants in the West are due to acute liver failure (1). Dilemmas surrounding timing of liver transplantation, prognosis and donor shortage highlight the need for disease modifying treatments in patients with ALF (3).

Acute-on-chronic liver failure (ACLF) on the other hand occurs in patients with established chronic liver disease or cirrhosis. Multiple stages of cirrhosis are recognized; in the compensated cirrhosis phase symptoms are modest and mortality low. Acute liver injuries result in hepatic and systemic inflammation in cirrhotic patients which then drive hepatic decompensation as manifest by the development of one or a combination of ascites, portal hypertensive gastrointestinal bleeding and or encephalopathy (4). The one year mortality increases from 3% in patients with compensated cirrhosis to 57% with acute decompensation; median patient survival falls from 12 years to only 2 years (4). There is a subgroup of patients that after acutely decompensating develop progressive extrahepatic organ failures on a background of severe systemic inflammation (5), this syndrome is referred to as ACLF (6) and has a short term mortality as high as 80% at 28 days (7); ACLF in fact represents the leading cause of mortality amongst patients with decompensated cirrhosis (7). Precipitating events for decompensation and eventually ACLF in patients with established cirrhosis include drugs, infections, bleeds and flares of the underlying disease (4).

There are differences and similarities in the pathophysiological responses that underpin the development of ALF and ACLF, both involve a complex interplay between damaged hepatocytes, liver sinusoidal endothelial cells (LSECs), resident and circulating immune cells that initiate and potentiate inflammation but also determine resolution. Platelets are key protagonists in a number of these processes. Known historically for their hemostatic function at sites of vascular injury, it is now well established that platelets participate actively in the immune response intimately linking thrombosis and inflammation, in a process described as thromboinflammation (8–11). Whilst platelets are clearly involved in acute liver inflammation (12, 13), the involvement is likely to be stage and location specific varying in patients with and without pre-existent liver disease (14, 15). Of particular interest in the treatment of liver diseases is defining coagulopathy sparing platelet activation pathways (12, 16). Here we discuss the dualistic roles of platelets in the initiation, amplification and resolution of acute liver injury and how this drives the development of ALF and ACLF.

## 2 Acute liver failure and acute-on-chronic liver failure

### 2.1 Acute liver failure

Sterile inflammation is a response to host cell damage in the absence of pathogens, a key step in restoration of homeostasis, however, becomes a pathological process in several causes of ALF including drug-induced liver injury, alcoholic hepatitis, non-alcoholic steatohepatitis and ischemia-reperfusion injury (17, 18). Central to this condition is the release and recognition of damage-associated molecular patterns (DAMPs) *via* pattern recognition receptors (PRRs). DAMPs are self-molecules with an ability to activate inflammation (19) including a number of different proteins, nucleic acids and mitochondrial components (20). They are released in inflammatory cell death, pyroptosis, necrosis and necroptosis (18, 21), in addition to non-inflammatory cell death during secondary necrosis of apoptotic bodies (22). PRRs are highly conserved receptors, originally discovered for their role in responding to pathogen associated molecular patterns (PAMPs), of which Toll-like receptors (TLRs) are best studied, responsible for promoting inflammation through cytokine production, chemokine production and expression of ligands involved in leucocyte adhesion and activation (17). In the liver, immune surveillance is performed by a number of resident and circulating leucocytes. Kupffer cells are particularly important sentinel macrophages of the liver participating in the immune response through detection of injury, leucocyte recruitment and mediate tissue repair (23). Importantly it has been demonstrated that platelets are the first cells to accumulate at sites of injury within the liver, thus generating interest into their role in the disease process (24).

### 2.2 Acute-on-chronic liver failure

In ACLF the main driver of widespread tissue injury is a systemic hyperinflammatory response (25), arising from massive release of inflammatory mediators including cytokines, chemokines, growth factors and bioactive lipid mediators. This leads to immune cell activation and subsequent immune-mediated tissue damage (26). Little is known about exact triggers however is likely to involve recognition of both PAMPs and DAMPs *via* PRRs. Approximately one-third of cases involve bacterial infections (27), attributed to bacterial translocation across the intestinal lumen (28). Inflammatory cell death, necroptosis and pyroptosis, is common in advanced liver disease triggering release of DAMPs propagating the immune response (29). The hyperinflammatory response often co-exists with innate immune dysfunction at humoral, physical and cell-mediated level (26). The condition is characterised by increased pro- and anti-inflammatory mediators (30), with the prevailing phenotype temporally and spatially dependent, although immunodeficiency has greater importance in advanced disease (31). Bernsmeier et al. (32), proposed a model whereby exaggerated inflammatory responses to DAMPs and PAMPs in cirrhosis promotes polarization of monocytes/macrophages to immunoregulatory phenotypes. In the presence of endothelial dysfunction, reverse migration of these regulatory cells leads to population expansion in distant organs and global immunosuppression. One might hypothesize that populations of exhausted leucocytes predispose patients to sepsis, driving expansion and activation of naïve innate immune cells potentiating cell damage. Dissecting these pathways, and elucidating the role of platelets, if any, is key to identify suitable therapeutic targets.

## 3 Platelets provide the bridge between inflammation and thrombosis in the liver

**Figure 1:**
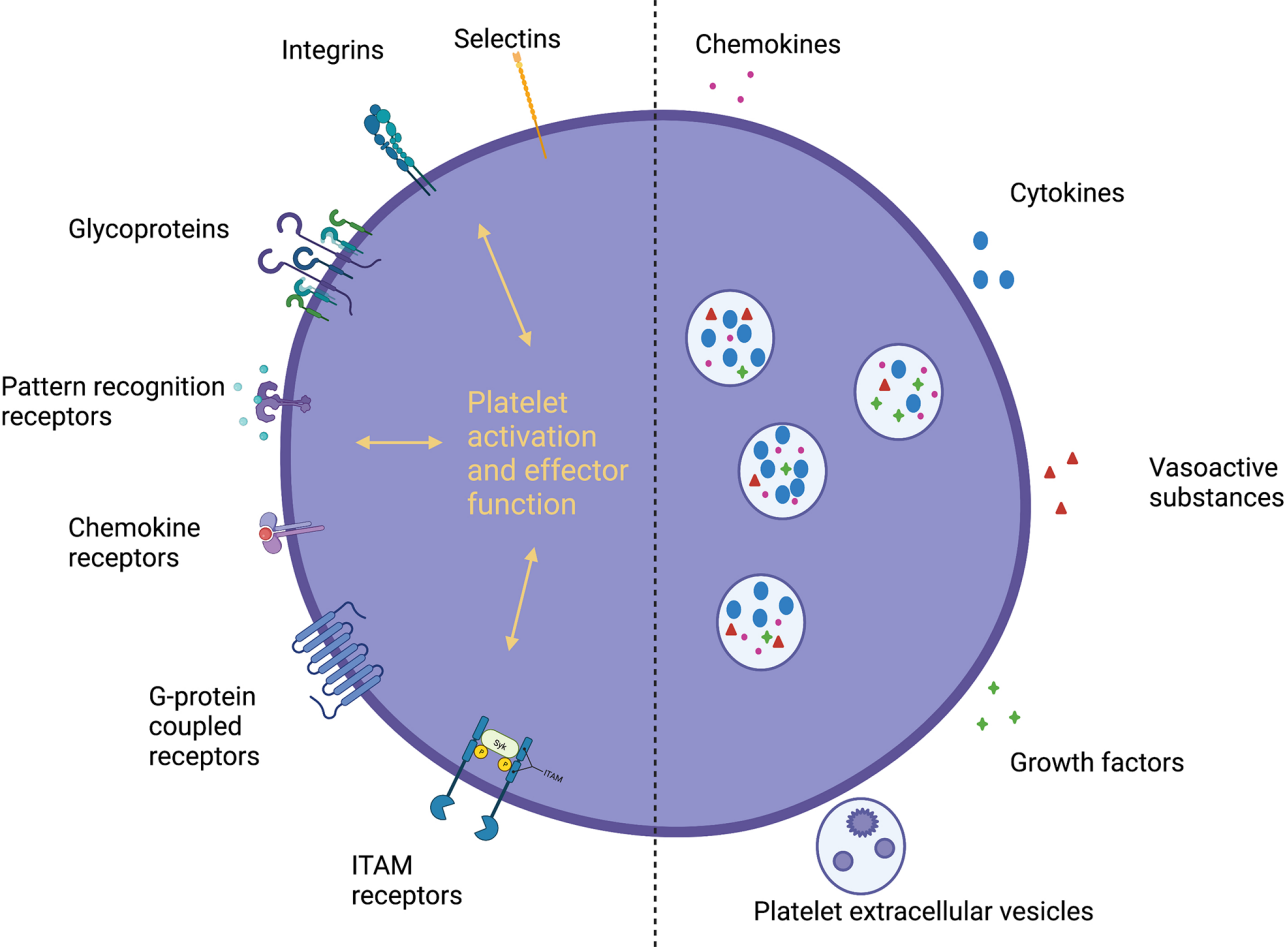
Platelet receptors and secreted mediators. Platelets express a diverse range of receptors and a large proteasome, secreting a variety of soluble mediators, facilitating direct and indirect interactions with immune cells, the endothelium and the extracellular matrix. *Pattern recognition receptors, PRRs; G-protein coupled receptors, GPCRs; Immunoreceptor tyrosine-based activation motif, ITAM; Platelet-derived extracellular vesicles, PEVs*.

Platelets possess a range of receptors and a diverse proteasome facilitating interactions with endothelial cells, immune cells, the extracellular matrix and other platelets (**Figure 1**) (9, 10, 33). Activation leads to platelet degranulation, through classical glycoprotein (GP) pathways at sites of vascular injury (9, 10) and more recently discovered alternative pathways (16), releasing cytokines, chemokines, vasoactive substances, growth factors and platelet-derived extracellular vesicles (PEVs) (containing microvesicles, exosomes and apoptotic bodies) (10, 34). Platelets exhibit dual roles in inflammation with pro- and anti-inflammatory effector functions, which are likely to be disease, organ and time-specific (10). They promote leucocyte recruitment and modulate effector functions through direct interactions (P-selectin-P-selectin glycoprotein ligand 1 (PSGL-1), GPIbα-macrophage-1 antigen (MAC-1, a complement receptor), GPIIbIIIa-MAC-1 through fibrinogen and CD40-CD40L pairings (35, 36)), chemokine/cytokine secretion and increasing vascular permeability (9, 10). They provide a link between innate and adaptive immunity, supporting antigen presentation and lymphocyte function (37). At sites of inflammation platelets also limit bleeding (38) through physical sealing and tightening of endothelial junctions (39). During injury resolution, platelets promote regeneration and homeostasis through chemokine, angiogenic factor and growth factor release (9). The interaction between platelets and leucocytes is bidirectional – leucocytes also promote platelet activation and, in turn, the coagulation cascade; this process is termed immunothrombosis (9). Initially recognized as a means to potentially limit pathogen spread and enhance clearance; there is now increasing recognition of the role microthrombi can play in liver pathobiology by inducing endothelial dysfunction and organ damage (13, 40).

**Figure 2:**
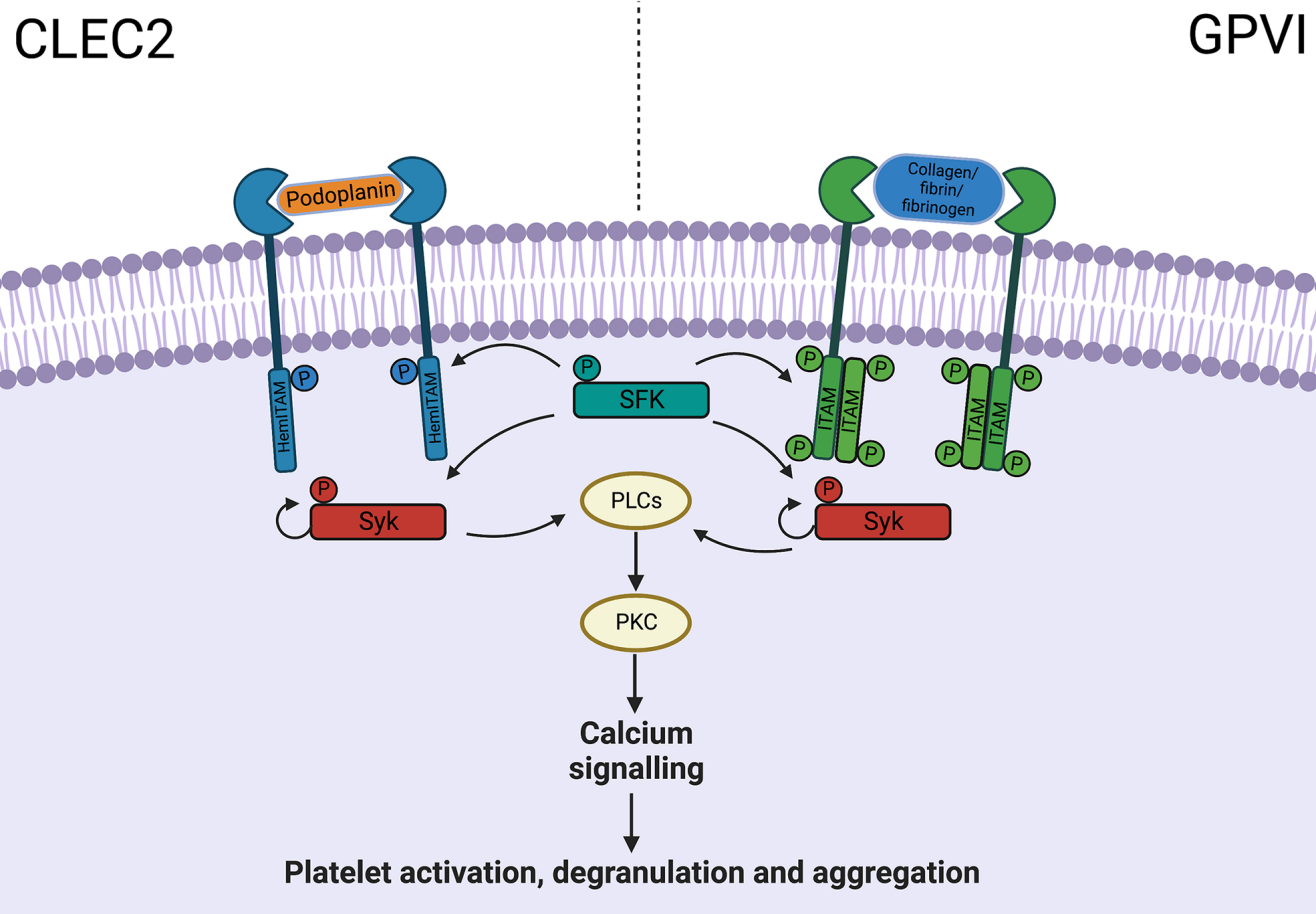
ITAM receptor downstream signalling pathway. CLEC-2 and GPVI share common downstream pathways, with activation leading to platelet aggregation and degranulation, through PLC, PKC and calcium signalling. *C-type lectin-like receptor 2, CLEC2; Glycoprotein VI, GPVI; Immunoreceptor tyrosine-based activation motif, ITAM; Phosphate, P; Src family kinase, SFK; Phospholipase C, PLC; Protein kinase C, PKC*.

The role for thromboinflammation is evident in conditions traditionally associated with thrombosis including atherosclerosis (41), deep vein thrombosis (42) and reperfusion injury after ischemic stroke (43). There is also increasing evidence for a protective role in sepsis, limiting tissue injury and promoting pathogen clearance (37, 44–48). Clinically, thrombocytopenia is associated with poor outcomes in sepsis (44, 46) and platelet transfusion improves bacterial clearance through macrophage recruitment (48). With a wide array of PRRs platelets aid with pathogen clearance during viral infection, but also exacerbate tissue injury in response to DAMPs (49). Interestingly platelets demonstrate plasticity in function, as evidenced in major trauma, emphasizing temporally and spatially diverse roles in the immune response (50). In the acute phase of injury platelets are poorly responsive to activation *ex vivo*, contributing to coagulopathy, followed by hyper-responsiveness exhibiting a pro-inflammatory and prothrombotic phenotype resulting in secondary organ damage (50). Pathways modulating platelet immune functions without impairing normal hemostasis are attractive therapeutic targets. Whilst this is particularly relevant in acute liver injury where patients can have an unpredictable coagulopathy, the bleeding diathesis in ALF is arguably an overstated concern but beyond the scope of this current review (51, 52). Immunoreceptor tyrosine-based activation motif receptors C-type lectin-like receptor 2 (CLEC2) and GPVI share similar downstream pathways involved in platelet activation (**Figure 2**) (16). CLEC2, activated by endogenous ligand podoplanin, is important in hemostasis although, promisingly, blockade does not produce a hemorrhagic phenotype (53). On the other hand, the podoplanin-CLEC2 axis appears to be vital in several models of thromboinflammation (16). In infection these receptors have both beneficial and detrimental roles (13, 45–47, 54). GPVI promotes neutrophil recruitment during pneumonia (54), whilst inflammatory macrophages promote platelet aggregation *via* CLEC2 leading to pathogenic thrombosis in peritoneal sepsis (13). In other mouse models targeting the podoplanin-CLEC2 axis may have a role in limiting immune activation (45–47).

In the liver, platelets promote hemostasis, fine tune the immune response through direct and indirect interactions and serve as a reservoir of biologically active substances (15). Unsurprisingly, platelet effector functions are complex and dualistic – often disease and stage specific (14).

### 3.1 Platelet interactions with the hepatic sinusoid endothelium

LSECs constitute a unique vascular bed with a powerful scavenger system and potent endocytic capacity, aptly placed to respond appropriately to an array of antigens balancing immune activation and tolerance (55). LSECs orchestrate the immune response but also interact with hepatocytes and hepatic stellate cells in the process of regeneration or fibrosis (55). The hepatic sinusoids are classically narrow, characterised by low shear stress and thus initial recruitment is often selectin independent. The sinusoids express minimal levels of selectins *in vivo* (56, 57). Platelet sequestration is observed within hepatic sinusoids in numerous models of inflammation including APAP-induced (12), non-alcoholic steatohepatitis (58), ischemia-reperfusion injury (59, 60) and viral hepatitis (61).

**Figure 3:**
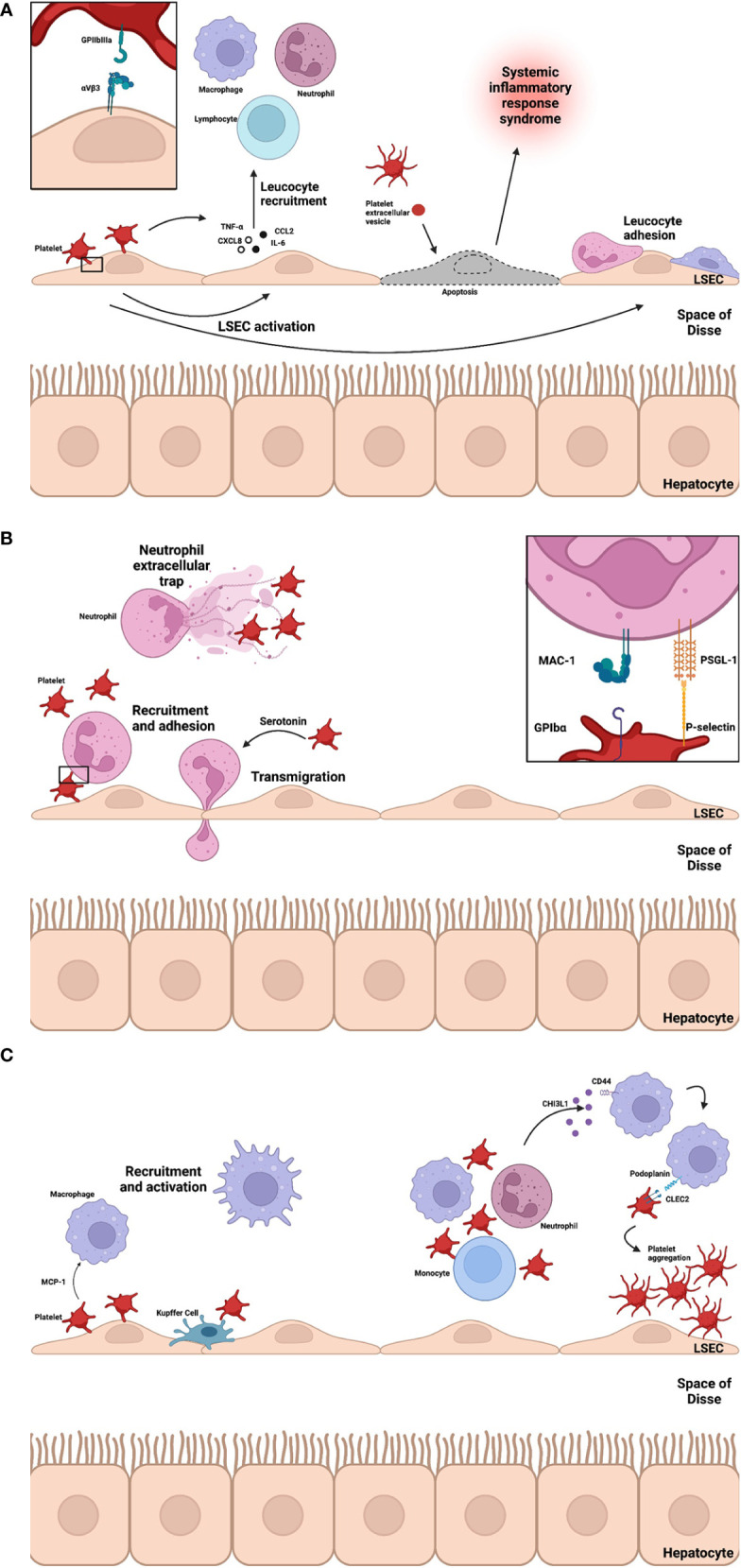
**(A-C)** Platelet interactions drive liver inflammation. At sites of inflammation platelets interact with LSECs leading to chemokine and cytokine (CXCL8, CCL2, IL-6 and TNF-α) secretion to facilitate recruitment of leucocytes. Platelet-LSEC interaction is partially integrin mediated (GPIIbIIIa and αVbeta3). PEVs rise rapidly at these sites and may induce apoptosis, driving systemic inflammation. Adherent platelets recruit neutrophils *via* P-selectin-PSGL-1 interactions and MAC-1 *via* GPIbα and platelet-derived 5-HT mediates neutrophil transmigration. The bidirectional relationship between platelet activation, aggregation and NETosis, a key effector function of neutrophils, is a key component of immunothrombosis and a driver of inflammation in ALF. During homeostasis KCs and platelets interact transiently through GPIb-vWF – A process of immune surveillance. Monocytes and macrophages are recruited to liver sinusoids through chemoattractant MCP-1. Recently, CHI3L1 release in models of ALF trigger podoplanin expression on macrophages which promote platelet aggregation and drive inflammation *via* CLEC2. *Liver sinusoidal endothelial cell, LSEC; Glycoprotein, GP; Tumour necrosis factor alpha, TNF-α; CXC motif chemokine ligand 8, CXCL8; interleukin 6, IL-6; CC ligand 2, CCL2; Systemic inflammatory response syndrome, SIRS; Platelet-extracellular vesicle, PEV; Neutrophil extracellular trap, NET; Serotonin, 5-HT; Macrophage-1 antigen, MAC-1; P-selectin glycoprotein ligand-1, PSGL-1; Acute liver failure, ALF; Kupffer cell, KC; Von Willebrand Factor, vWF; Monocyte chemoattractant protein-1, MCP-1; chitinase-3 like protein-1, CHI3L1; C-type lectin-like receptor 2, CLEC2*.

Platelet-endothelial interactions are also bidirectional and vary between type of injury encountered. Due to the diverse range of receptors, surface ligands and ability to secrete soluble mediators, both cell types participate in recruitment, adhesion, and activation of immune cells (**Figure 3A**). *In vitro* studies have demonstrated platelet adhesion is, in part, integrin mediated (through GPIIbIIIa and αVβ3) (62). Platelet adhesion triggers CXCL8 and CCL2 production by endothelial cells, promoting leucocyte recruitment. In a model of liver ischemia-reperfusion injury, LSECs are highly susceptible during cold preservation (63). Depletion of adenosine triphosphate (ATP) impairs transmembrane active ion transport leading to cellular swelling and mitochondrial dysfunction (64). On reperfusion reactive oxygen species production depletes free radical scavengers, leading to activation with increased expression of P-selectin (60). Upregulation of P-selectin promotes platelet adhesion, activation and LSEC apoptosis (65). In a bile duct (BDL) model of cholestatic liver injury of mice, platelet accumulation promotes leucocyte sequestration and hepatocyte damage in a partially p-selectin dependent manner (66). In this model, the role of LSEC podoplanin expression has been explored (67). Podoplanin expression is increased in BDL-treated mice and those pre-treated with anti-CLEC2 antibodies had reduced hepatic inflammation and subsequent fibrosis. The authors hypothesized platelet-derived serotonin (5-HT) released on activation of CLEC2 was responsible for reduced injury and promoting regeneration. Plasma serotonin was found to be raised in untreated BDL-mice. PEVs have been shown to induce endothelial cell apoptosis in models of sepsis (68). In a study of patients presenting with ALF (50% APAP-induced), increased levels of circulating microparticles were associated with presence of systemic inflammatory response syndrome, high-grade hepatic encephalopathy and death or requirement for liver transplantation (69). This may implicate PEVs in driving inflammation in ALF or simply highlight their utility as a marker of systemic inflammation. In ischemia-reperfusion injury, PEV levels increase rapidly at initiation of injury (70) and inhibition of microparticle release has been shown to reduce degree of injury (71). Endothelial cells interact with resident and migrating leucocytes to promote platelet adhesion and activation. In a model of ischemia-reperfusion injury, migrating CD4 T cells were shown to interact with endothelial cells to promote platelet adhesion (72) and Kupffer cells produced a similar effect *via* tumor necrosis factor (TNF)-α secretion (73). Bidirectional communication promotes a self-perpetuating cycle of inflammation leading to significant immune-mediated damage. These murine and human data reveal how platelets modulate the inflammatory landscape in acute liver injury in non-fibrotic livers to potentially drive ALF.

#### 3.1.1 Platelets driven thromboinflammation impairs the hepatic microcirculation

Evidence for thromboinflammation mediated liver damage in acute liver injury has clearly been demonstrated in viral models of murine hepatitis. Activated platelets in these models reduce sinusoidal blood flow through the secretion of vasoactive mediators including 5-HT, which then drives the development of inflammatory intrahepatic microthrombosis. Thromboinflammation thus directly delays effector cell recruitment reducing viral clearance and enhancing liver damage (74). During homeostasis, surface expression of CD39 on LSECs cleave ATP and adenosine diphosphate to adenosine monophosphate limiting platelet activation (75). In reperfusion injury, for example during ischemic hepatitis, CD39 expression is reduced promoting platelet activation *via* ATP. Injury is again potentiated through increased vascular tone and reduced blood flow from platelet-derived thromboxane A2 and 5-HT (59). Historic models reveal that during acute liver injury in rats endothelial damage causes the deposition of platelet rich thrombi within hepatic sinusoids (76). These models demonstrate that thromboinflammation disrupts hepatic microcirculation (77); reduced sinusoidal blood flow exacerbates hepatocyte dysfunction, increases DAMP expression and amplifies inflammation. These data provide a cogent explanation to how platelet driven thromboinflammation can drive both *de novo* acute liver failure for instance in virus induced ALF but also decompensation and ACLF in patients who contract a viral infection or suffer an ischemic hepatitis on a background of established liver cirrhosis.

Non-selective beta blockers are used in cirrhosis to reduce portal pressure. They also exert beneficial non-hemodynamic effects in relation to bacterial translocation, particularly reducing incidence of ACLF secondary to bacteria-induced systemic inflammation (CANONIC study) (78). Recently reduced von Willebrand Factor (vWF) levels, as a marker of endothelial dysfunction, were identified as a possible marker of non-hemodynamic non-selective beta blocker effect (79). This may illustrate a link between endothelial dysfunction, platelets and bacteria-induced systemic inflammation in ACLF. More recently, examination into predictors of ACLF development during acute decompensation identified severity of inflammation as a key determinant of the development of portal venous thrombosis; again highlighting the link between inflammation and thrombosis in the context of liver cirrhosis (80). Further evidence for the role of immunothrombosis is observed in severity of decompensated cirrhosis (81). Here, portal and hepatic vein sampling was performed in patients undergoing transjugular intrahepatic portal systemic shunt insertion and demonstrated higher markers of platelet activation, lipopolysaccharide levels and inducible nitric oxide synthetase in the portal vein. This correlated with clinical disease severity, linking bacterial translocation and platelet activation with progression of disease. The specific molecular endothelial triggers for platelet activation within the liver and how this varies when comparing cirrhotic to non-cirrhotic livers merits further investigation.

### 3.2 Platelet-neutrophil interactions

Neutrophils are one of the key effector cells in the innate immune system responsible for driving inflammation and tissue injury in the liver (82–86). They are essential to host defense (87) but can also contribute to indiscriminate tissue injury and are responsible for cell damage in many diseases (88, 89). Neutrophil recruitment and activation is evident in a multitude of acute liver injuries which can drive acute liver failure including drug-induced liver injury (82), and ischemia-reperfusion injury (86) but also ACLF including viral hepatitis (83), alcoholic hepatitis (84), and non-alcoholic steatohepatitis (85). Neutrophils infiltrate the site of liver injury within minutes to hours *via* stimulated LSECs involves a process of selectin-mediated rolling, integrin-mediated firm adhesion followed by transendothelial migration in a well-described recruitment cascade (89). Recruitment to the liver during inflammation is influenced by resident Kupffer cells (90–92), LSECs (93) and stromal cells (94). Necrotic cells release necrotaxis signals including mitochondrial formylated peptides to facilitate precise homing (91). Neutrophils migrate through intravascular channels and exhibit swarming behaviour at the site of injury (91). In addition to initiating and propagating inflammation, neutrophils promote resolution and a return to homeostasis in focal thermal (24), APAP- (12) and carbon tetrachloride-induced (95) liver injury through clearance of cellular debris, production of extracellular matrix and normal revascularization. During ACLF neutrophils exhibit an exhausted phenotype – characterised by high levels of activation (96) but impaired core functions including phagocytosis, reactive oxygen specifies production and degranulation (97). Lower CXCR1/2 expression on neutrophils, a key chemotactic receptor, predicted poor outcome in hepatitis B-virus (HBV) related ACLF (98). On the other hand, neutrophil-to-lymphocyte ratio has been shown to be an independent predictor of prognosis in HBV ACLF (99). Interestingly, patients with ratios ≥3 had lower mortality, but those with >6 were at greater risk of mortality in ACLF.

#### 3.2.1 Platelets facilitate neutrophil recruitment to the liver

Platelets contribute to neutrophil recruitment and activation through ligand-receptor interactions and chemokine secretion (**Figure 3B**) (10, 100, 101). Initial interactions between platelet P-selectin and neutrophil PSGL-1 are critical for recruitment, activation of MAC-1 and LFA-1 (lymphocyte function-associated antigen 1, an integrin expressed on leucocytes) and release of neutrophil extracellular traps (NETs) (102, 103). The importance of platelet-neutrophil interactions *via* p-selectin is illustrated by improved survival of p-selectin deficient mice in an ischemia-reperfusion injury model of acute liver injury (104). MAC-1 allows direct binding with platelets *via* GPIbα and indirectly to GPIIbIIIa *via* fibrinogen (105). Recruitment is further amplified through secretion of cytokines, chemokines and growth factors including platelet factor 4 (PF4), interleukin(IL)-1, RANTES, beta-thromboglobulin, platelet-derived growth factor (PDGF), platelet-activating factor, CXCL7, migration inhibiting factor, thromboxane A2 and 5-HT (10). The interaction between platelets and neutrophils in the liver has been observed in models of sterile injury (24). Using intravital confocal microscopy, platelets were observed to line the endothelium at sites of focal injury facilitating neutrophil rolling through GPIIbIIIa dependent mechanisms.

ACLF, often triggered by sepsis, rapidly leads to multiorgan dysfunction (6), and platelets may contribute to development of the systemic inflammatory response. In a mouse model of endotoxemia-mediated acute lung injury, P-selectin-PSGL-1 interactions were investigated (106). Administration of PSGL-1 blocking antibody reduced recruitment of neutrophils, platelet-neutrophil aggregates, lung injury and survival. It may be of interest to assess the role of this interaction in models of ALF and ACLF. Whilst platelet-neutrophil interactions may exacerbate injury, recent work may highlight a beneficial role for platelet-derived 5-HT in neutrophil recruitment to sites of inflammation. Giovanni et al. (107), demonstrate 5-HIAA, a metabolite of platelet-derived 5-HT, mediates neutrophil recruitment and transmigration to inflamed tissues *via* GPR35. Here, treatment with serotonin inhibitors diminished recruitment of neutrophils and clearance of peritoneal bacteria.

How platelets influence neutrophil recruitment and final phenotype within the damaged liver and whether parallels from data in other organs can be extrapolated to the liver is key to develop rational antiplatelet therapies in liver disease.

#### 3.2.3 Platelet driven NETosis: Key to both ALF and ACLF?

Neutrophils release NETs as an antimicrobial effector mechanism (108, 109). These consist of a fibrous mesh of decondensed DNA mixed with a number of nuclear and granular proteins capturing and neutralizing microbes in an attempt to prevent dissemination (110). In the liver, NETs are released in response to infection, ischemia and sterile inflammation (111). Whilst providing an important role in pathogen defense, NETs are cytotoxic towards host cells (112). The neutrophil-platelet-NET axis is a complex interaction whereby platelet-mediated recruitment and activation of neutrophils at sites of inflammation triggers NET release (113). NET release in response to platelet activation is integrin- (114) and potentially selectin-mediated (115). Histone and polyphosphate entities within the NET matrix subsequently activate platelets through TLRs and directly activate coagulation within the bloodstream (116, 117). The neutrophil-platelet-NET axis illustrates the important role for link between infection, inflammation and thrombosis enhancing host immunity (118). In models of sepsis (116, 119), and recently in a galactosamine hydrochloride and lipopolysaccharide model of ALF (120), NETs contribute to tissue damage. Importantly, inhibition of NET generation or DNAse to breakdown NET reduces observed collateral tissue damage (116, 119). Cell-free DNA, often referred to as a NET marker (albeit somewhat non-specific) is associated with mortality in ACLF though the link with the more specific myeloperoxidase-DNA was not established (121). In a study by von Meijenfeldt et al (122), 676 patients with ALF were recruited from the U.S ALF Study group. Forty-six percent had APAP-induced ALF. Cell-free DNA and myeloperoxidase-DNA complexes were measured in comparison to healthy controls and tissue obtained at liver transplantation was stained for NETs in 20 patients. Levels of cell-free DNA and myeloperoxidase-DNA complexes were 7.1-fold and 2.5-fold higher than healthy controls respectively. High cell-free DNA was not associated with mortality. Myeloperoxidase-DNA levels were 30% higher in patients with ALF who died or required urgent liver transplant. The observed differences between ALF and ACLF may be explained, in part, by innate immune cell dysfunction in ACLF. NETs may represent an attractive therapeutic target to reduce immunothrombosis and cytotoxic cell damage, however this needs to be carefully balanced with loss of beneficial immune function. In animal models of sepsis, disruption of NET formation reduced liver injury and microcirculation thrombosis without impairing bacterial clearance (123–125). More research is required to accurately measure NET formation (108) and their impact in different models of liver injury.

### 3.3 Platelet-monocyte/macrophage interactions

Monocytes and macrophages have a critical role in homeostatic immune mechanisms, immune-mediated liver injury, fibrosis and regeneration (126). Infiltrating the site of injury 24-48hrs after neutrophils, monocytes perform diverse functions during ALF including inflammatory mediator release, clearance of dead cells, stimulation of the extracellular matrix and parenchymal regeneration (18). Distinct subsets of monocytes possess predominately inflammatory or anti-inflammatory phenotypes (**Table 1**) (18). Differentiation into macrophages is also an important function (127, 128). Platelet interactions are evident in immune surveillance functions of the liver, initiation of inflammation, recruitment of monocytes and polarisation to a pro-inflammatory macrophage profile (**Figure 3C**) (14, 15). Monocytes, sharing common regulatory and effector properties with neutrophils, are recruited to inflamed endothelium by platelets in a similar fashion (15). Efficient monocyte recruitment requires specific stimuli, namely monocyte chemoattractant protein 1 (MCP-1) (10).

**Table 1:** Monocyte subsets.

Human Subsets		Mouse subsets	
Classical	CD14^++^CD16^-^	Inflammatory	CCR2^hi^, CX_3_CR1^lo^
Intermediate	CD14^++^CD16^+^	Anti-inflammatory	CX_3_CR1^hi^, CCR2^lo^
Non-classical	CD14^+^CD16^++^		

Distinct subsets of monocytes exist, characterised by surface receptor expressions and possess differing trafficking patterns and effector functions.

The interaction between Kupffer cells and platelets is an important step in initial pathogen detection. Platelets survey macrophages through transient GPIb-vWF interactions during homeostasis (129). In the presence of blood-borne bacteria, sustained platelet-macrophage interactions are observed through vWF-GPIIb/IIIa encasing the bacterium and facilitating clearance. Increasing evidence is suggesting that the podoplanin-CLEC2 axis is central to platelet interactions with macrophages. Recently, Shan et al. (130), identified a novel interaction between platelets and macrophages potentiating APAP-induced liver injury through chitinase-3 like protein-1 (CHI3L1). CHI3L1 is a soluble protein released by multiple immune cells and found to be raised in a range of liver disease (131). In this study, CHI3L1 interaction *via* CD44 on macrophages upregulated podoplanin expression and subsequent platelet aggregation *via* CLEC2. In this model, disruption of the pathway at the level of CHI3L1 and podoplanin-CLEC2 greatly inhibited liver injury after APAP administration. In the model of APAP-induced liver injury (132), platelet depletion greatly reduced tissue damage and the study by Shan et al (130), highlights one potential pathway underlying this – its role in the other models of liver injury are yet to be determined.

#### 3.3.1 Divergent roles in ALF and ACLF – Polarisation, plasticity and immunoparesis

Monocytes participate in early stages of the innate immune response to acute liver injury through cytokine production, antigen presentation and polarisation to inflammatory macrophages (30). Plasticity in response to the local microenvironment is demonstrated with anti-inflammatory monocytes appearing 12-24 hours later promoting resolution *via* IL-10, transforming growth factor beta (TGF-β) and vascular endothelial growth factor (VEGF) (133–136). This arises from recruitment and *in situ* reprogramming (136). Apart from monocyte differentiation, specific macrophage populations resident to the peritoneal cavity, characterised by GATA6 expression, are recruited to the inflamed liver to assist in liver repair (137–140). This appears to be mediated by ATP release and exposed hyaluronan (137, 138). Recently, Jin et al. (139), demonstrated through dual recombinase mediated genetic GATA6+ lineage tracing, macrophages are only recruited to surface of liver during carbon tetrachloride-induced liver injury, questioning a potential role in ALF.

In ACLF, there is evidence of dampened function, characterised by reduced human leukocyte antigen (HLA)-DR expression, correlated with high mortality rates and increased prothrombin time (31). Underlying immunoparesis in ACLF, there is also expansion of several monocyte populations including MERTK^+^ (32), monocytic myeloid-derived suppressor cells (141) and intermediate CD14^++^CD16^+^ (142) with classical monocytes also exhibiting impaired function, characterised by reduced TLR2/4 expression, phagocytic activity and upregulation of genes related to dampened immune response (142). Monocyte/macrophage polarisation and plasticity is influenced by platelet activity. *In vitro* studies by Lee et al. (143), demonstrated adenosine diphosphate-activated platelets induced CD16 expression on CD14+CD16- monocytes from platelet-derived TGF-β and monocyte-derived IL-6. These monocytes preferentially differentiated towards M2 macrophages expressing CD163 and MerTK. It may be postulated that in platelet-monocyte interactions in advanced cirrhosis contributes to immunoparesis through expansion of MerTK macrophages precipitating widespread inflammation in ACLF. In contrast, *in vitro* lipopolysaccharide-treated monocytes co-incubated with platelets are skewed from an M2 towards a pro-inflammatory M1 phenotype demonstrating increased TNF-α expression, improved bacterial phagocytic activity and reduced healing capability (48). *In vivo* platelet transfusion increased inducible nitrous oxide synthase-expressing macrophages, improving bacterial clearance and survival in septic mice. In both of these settings, blockade of CD11b-GPIb interaction abolished the effect. The effect of platelet-macrophage interactions in liver disease is not limited to the hepatic environment as in a murine model of acute liver injury monocyte-platelet aggregates modulated microglial activation and drove the development of sickness behaviors in TLR4-dependent pathways (140).

Platelet interactions with monocytes and macrophages are complex and diverse. Given the distinct cellular niches that exist within fibrotic livers (144), studying spatio-temporal platelet driven immunothrombosis in acute and chronic liver disease and how this influences macrophage phenotype and function in liver inflammation remains an exciting avenue to study.

## 4 The role of platelets in resolution of inflammation

**Figure 4:**
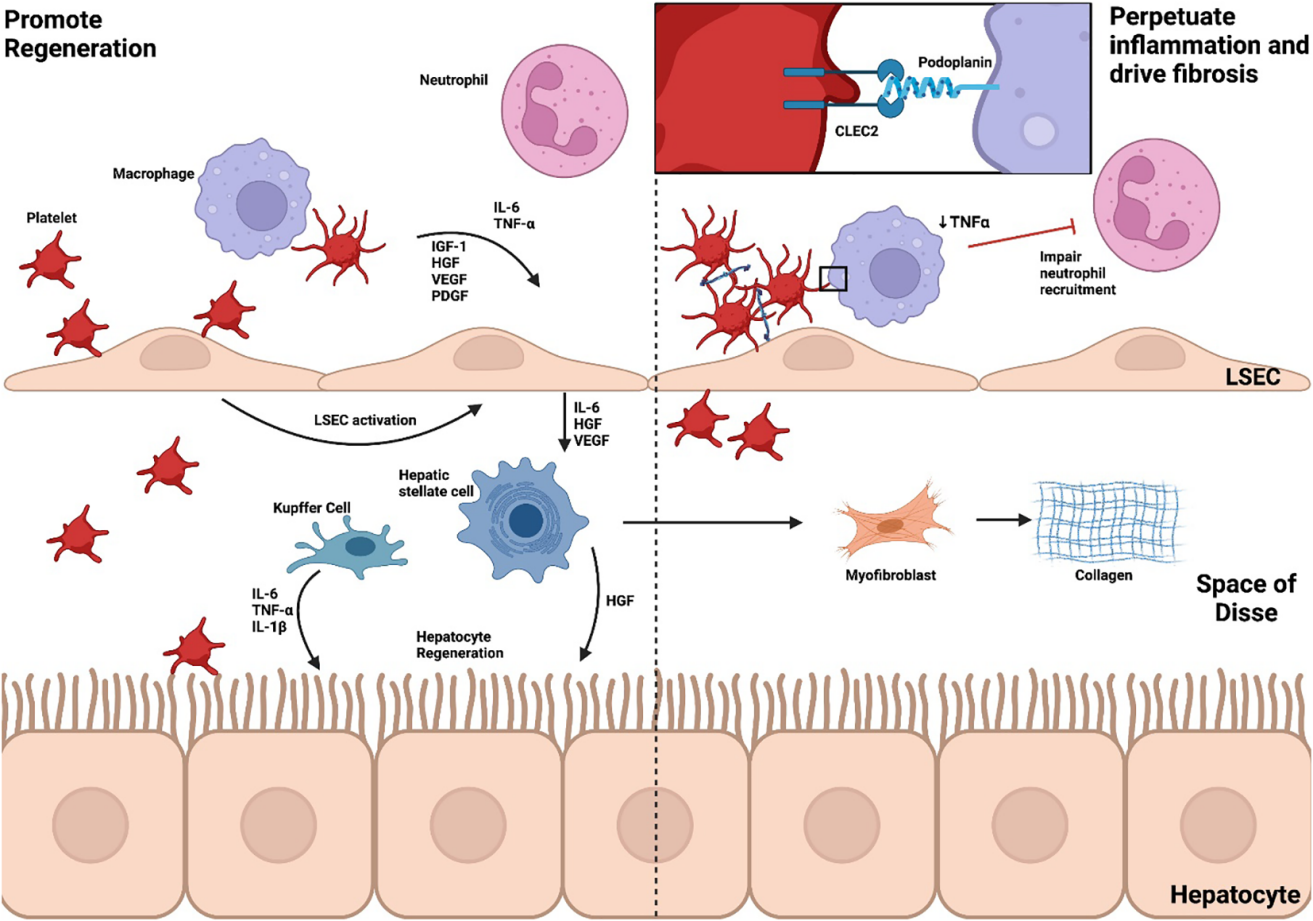
Platelets promote regeneration, perpetuate inflammation and drive fibrosis. The role of platelets in the resolution of inflammation is dualistic, potentiating immune-mediated damage, promoting regeneration and driving fibrosis. Through CLEC2, platelets activate LSECs to produce IL-6, TNF-α, HGF and VEGF which, in turn, triggers HGF release from HSCs to stimulate hepatocyte regeneration. HSC activation also leads to myofibroblast differentiation and collagen synthesis. Neutrophils, critical to resolution of inflammation, are also recruited through cytokine and chemokine release. In an APAP model of ALF, however, platelet-macrophage interactions through CLEC2 reduce neutrophil recruitment and perpetuate inflammation. *Liver sinusoidal endothelial cell, LSEC; Kupffer cell, KC; Hepatic stellate cell, HSC; interleukin, IL; Tumour necrosis factor alpha, TNF-α; Insulin-like growth factor-1, IGF-1; Hepatocyte growth factor, HGF; Vascular endothelial growth factor, VEGF; Platelet-derived growth factor, PDGF; C-type lectin-like receptor 2, CLEC2; Acetaminophen, APAP; Acute liver failure, ALF*.

Platelets play a provocative role in the resolution of inflammation with the ability to potentiate immune-mediated damage, promote regeneration and drive fibrosis (**Figure 4**). This complex relationship may reflect limitations in models used to study liver inflammation, represent gaps in our knowledge or identify roles for platelets that vary throughout and in different types of injury.

During liver regeneration, hepatocyte proliferation is controlled by a multitude of extracellular signals including cytokine, growth factor and metabolic pathways (15). TNF-α and IL-6 are cytokines central to regulation of liver regeneration whilst growth factors such as hepatocyte growth factor, endothelial growth factor, Insulin-like growth factor-1 and PDGF drive cell cycle progression (145). Platelets accumulate within the space of Disse rapidly after partial hepatectomy and have an active role in hepatic regeneration (146–148). Such a role for platelets is also appreciated in the clinical setting in a recent meta-analysis of 3966 patients (149). In this study preoperative thrombocytopenia constituted a significant risk factor for post-hepatectomy liver failure. Platelets mediate regeneration through interactions with LSECs, Kupffer cells and hepatocytes. These interactions are facilitated by direct contact and platelet-derived soluble mediators including hepatocyte growth factor, insulin-like growth factor 1, PDGF, VEGF, 5-HT, adenosine diphosphate and ATP (150). Downstream cascades key to these pathways include TNF-α/NF-kB, Il-6/STAT3, phosphatidylinositol 3-kinase (PI3K)/Akt and ERK1/2 (15, 151).

LSECs produce mitotic substances, specifically IL-6, hepatocyte growth factor and VEGF (152). Direct adhesion between platelets and LSECs induces IL-6 release, in turn, leading to hepatocyte growth factor secretion from hepatic stellate cells promoting hepatocyte regeneration *in vitro* (153). This is likely controlled through podoplanin-CLEC2 signalling (154) and expression of vWF (155). Soluble mediators released by activated platelets including TGF–β1 (156) and sphingosine-1-phosphate (157) are also sufficient to support IL-6 production by LSECs.

Kupffer cells are an important source of regenerative cytokines TNF-α, IL-6 and IL-1β (126). Depletion of Kupffer cells impairs hepatocyte proliferation during liver regeneration in cholestatic injury (158), alcohol-induced injury (159) and partial hepatectomy (147). Platelets contribute to regeneration through promoting TNF-α production by Kupffer cells, however utilize alternate pathways in Kupffer cell depleted environments (147).

Platelet-derived 5-HT is an important mediator of liver regeneration, most likely through production of growth factors at the site of injury (160, 161). Recently a multi-center trial has demonstrated that perioperative use of selective serotonin reuptake inhibitors and serotonin noradrenaline reuptake inhibitors is associated with adverse outcomes after hepatic resection further supporting a role for 5-HT (162).

Within the space of Disse, platelets are also able to interact with hepatocytes directly. Internalization of platelets and platelet-like particles followed by horizontal transfer of mRNA has also been demonstrated to contribute to hepatocyte proliferation (163). Linking coagulation and inflammation, liver-specific tissue factor release promotes platelet accumulation through fibrin(ogen) deposition, facilitating resolution after partial hepatectomy (164).

Whilst it is generally accepted that platelets are able to promote liver regeneration through a variety of mechanisms, there is emerging evidence implicating them in delaying resolution (12, 77). Neutrophils are not only central drivers of inflammation but also promote resolution during sterile injury (24). Recently our group demonstrated signalling *via* podoplanin-CLEC2 between platelets and inflammatory macrophages reduced TNF-α secretion and subsequent neutrophil recruitment to facilitate resolution of inflammation in APAP toxicity (12). Moreover, increased vWF deposition and impaired clearance has been attributed to persisting platelet accumulation in APAP-induced liver disease (77). In this model, deficiency or inhibition of vWF accelerated resolution. These results add a further layer of complexity to our understanding highlighting that targeting platelets may be temporally sensitive but also vary in different models of liver injury (12, 77, 155). Partial hepatectomy is the most commonly studied model of liver regeneration, however pathways involved in injury and resolution vary in other models (165). Thus, further investigation is required in different models of ALF and ACLF to elucidate specific roles of platelets and identify potential therapeutic targets.

## 5 Conclusions

Platelets play a vital role at all stages of acute liver injury through direct and indirect interactions with immune cells, stromal cells and the endothelium. Their involvement is both beneficial and detrimental. On one hand, they can intelligently sense and appropriately respond to pathogens, recruit leucocytes and promote regeneration at the resolution of inflammation. On the other, they exaggerate immune-mediated tissue injury, worsen hepatocyte dysfunction through microthrombi formation and delay mechanisms of resolution. Pathways controlling platelet effector function, such as the podoplanin-CLEC2 axis, represent an attractive therapeutic target however the disease-specific and temporal roles of platelets need to be carefully dissected in order to develop effective disease modifying treatments.

## Author contributions

SM – conceptualization, writing of original draft and editing. AC – overall supervision of article. All authors contributed to the article and approved the submitted version.

## Acknowledgments

All figures created with BioRender.com

## Conflict of interest

Author AC is recipient of a Wellcome trust clinical fellowship and an NIHR clinical lectureship. AC has received consultation fees from Principia biopharma now part of Sanofi.

The remaining author declares that the research was conducted in the absence of any commercial or financial relationships that could be construed as a potential conflict of interest.

## Publisher’s note

All claims expressed in this article are solely those of the authors and do not necessarily represent those of their affiliated organizations, or those of the publisher, the editors and the reviewers. Any product that may be evaluated in this article, or claim that may be made by its manufacturer, is not guaranteed or endorsed by the publisher.
